# TWIK-1 BAC-GFP Transgenic Mice, an Animal Model for TWIK-1 Expression

**DOI:** 10.3390/cells10102751

**Published:** 2021-10-14

**Authors:** Osung Kwon, Hayoung Yang, Seung-Chan Kim, Juhyun Kim, Jaewon Sim, Jiyoun Lee, Eun-Mi Hwang, Sungbo Shim, Jae-Yong Park

**Affiliations:** 1 Department of Integrated Biomedical and Life Science, Graduate School, Jiyoun Lee Korea University, Seoul 02841, Korea; osung@korea.ac.kr (O.K.); 216008@kist.re.kr (S.-C.K.); KJh9261@gmail.com (J.K.); shgkyniu@gmail.com (J.S.); hiu03061@naver.com (J.L.); 2 Department of Biochemistry, Chungbuk National University, Cheongju 28644, Korea; hayoung.yang10@gmail.com; 3 Center for Functional Connectomics, Korea Institute of Science and Technology (KIST), Seoul 02792, Korea; emhwang@kist.re.kr; 4 BK21FOUR R&E Center for Learning Health Systems, Korea University, Seoul 02841, Korea

**Keywords:** TWIK-1, bacterial artificial chromosome transgenic mouse, dentate gyrus, lateral entorhinal cortex, cerebellum

## Abstract

TWIK-1 is the first identified member of the two-pore domain potassium (K2P) channels that are involved in neuronal excitability and astrocytic passive conductance in the brain. Despite the physiological roles of TWIK-1, there is still a lack of information on the basic expression patterns of TWIK-1 proteins in the brain. Here, using a modified bacterial artificial chromosome (BAC), we generated a transgenic mouse (Tg mouse) line expressing green fluorescent protein (GFP) under the control of the TWIK-1 promoter (TWIK-1 BAC-GFP Tg mice). We confirmed that nearly all GFP-producing cells co-expressed endogenous TWIK-1 in the brain of TWIK-1 BAC-GFP Tg mice. GFP signals were highly expressed in various brain areas, including the dentate gyrus (DG), lateral entorhinal cortex (LEC), and cerebellum (Cb). In addition, we found that GFP signals were highly expressed in immature granule cells in the DG. Finally, our TWIK-1 BAC-GFP Tg mice mimic the upregulation of TWIK-1 mRNA expression in the hippocampus following the injection of kainic acid (KA). Our data clearly showed that TWIK-1 BAC-GFP Tg mice are a useful animal model for studying the mechanisms regulating TWIK-1 gene expression and the physiological roles of TWIK-1 channels in the brain.

## 1. Introduction

Two-pore domain potassium (K2P) channels contribute to background potassium conductance in cells and control the resting membrane potential (RMP) as well as neuronal excitability [1]. The 15 isoforms in the K2P channel family are modulated by diverse physical and chemical stimuli, including membrane stretch, pH, temperature, polyunsaturated fatty acids, hormones, and neurotransmitters [1]. Within the K2P channel family, TWIK-1 (tandem of pore domains in weak inward rectifying K^+^ channel 1, often referred to as KCNK1 or K2P1) was initially cloned from a human kidney cDNA library and expressed in various tissues, including the kidney, heart, and brain [2]. However, the electrophysiological properties and functional roles of TWIK-1 in the brain are poorly understood, since TWIK-1 exhibits low or non-measurable TWIK-1 currents in heterologous expression systems [3,4].

Interestingly, TWIK-1 knockout mice show defects in phosphate transport in the proximal tubule and water transport in the medullary collecting duct of the kidney, as well as hyperpolarization in the resting membrane potential of pancreatic cells [5,6]. In addition, we recently reported that TWIK-1 contributes to maintaining proper neuronal excitability and astrocytic passive conductance in the mouse hippocampus [7,8,9,10,11]. Deficiency of TWIK-1 induced with specific short hairpin RNA (shRNA) results in increased RMP, which increases the excitability of hippocampal dentate gyrus (DG) granule neurons [7,8]. Additionally, TWIK-1 is a key component of astrocytic passive conductance, which is important for potassium buffering [9,10,11,12]. Therefore, these previous studies strongly suggest the important physiological roles of TWIK-1 channels in various tissues including the brain.

Functional studies of TWIK-1 have been intensively conducted in the hippocampal region of the mouse brain [7,8,9,11]. However, previous in situ hybridization data for K2P families showed TWIK-1 expression in various regions of the mouse brain [13,14]. Therefore, detailed studies of TWIK-1 expression with cell-type-specific levels are necessary to understand the cellular TWIK-1 functions in various regions of the brain. To examine TWIK-1 expression at the cellular level in the brain, we generated a transgenic mouse (Tg mouse) line that expresses green fluorescent protein (GFP) under the control of the mouse TWIK-1 promoter (TWIK-1 BAC-GFP Tg mouse). In general, since modified bacterial artificial chromosomes (BACs) carrying ~150 kilobases (kb) of genomic DNA are generally used, BAC transgenic mice are one of the most widely used animal models for the correlation of gene expression with cell type to analyze gene expression in neuroscience research [15,16].

Here, we report the generation of TWIK-1 BAC-GFP Tg mice to examine TWIK-1 gene expression in the mouse brain at the cellular level. Immunohistochemical analysis showed that the distribution of GFP and endogenous TWIK-1 expression overlapped. Our data revealed that TWIK-1 is highly expressed in the DG, striatum, peri-amygdala area, reticular thalamic nucleus, lateral entorhinal cortex (LEC), and cerebellum (Cb). Moreover, the intraperitoneal injection of kainic acid (KA) induced upregulation of GFP and endogenous TWIK-1 expression in the hippocampus of TWIK-1 BAC-GFP Tg mice. Overall, TWIK-1 BAC-GFP Tg mice could provide detailed gene expression information on TWIK-1, which is useful for future studies on the role of TWIK-1 in the brain.

## 2. Materials and Methods

### 2.1. Generation and Maintenance of Transgenic Mice

**Figure 1:**
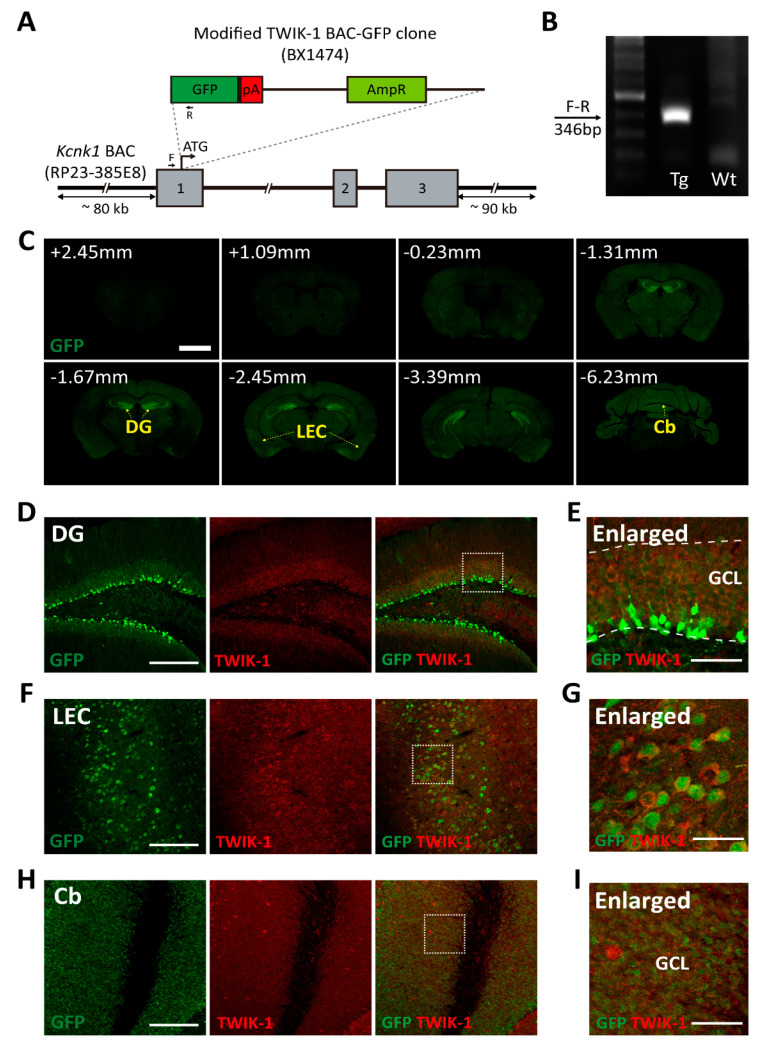
Generation of TWIK-1 BAC-GFP Tg mice. (**A**) Schematic design of the modified *Kcnk1* BAC vector. GFP coding sequence was inserted just before the start codon of *Kcnk1*. (**B**) Genotype PCR results for the designed primer pair in A. (**C**) Representative macroscopic GFP fluorescent images in the adult brain (P56 age) from TWIK-1 BAC-GFP Tg mice. Upper left numbers indicate the relative position of the brain slice from bregma. Strong GFP expression was identified in DG, LEC, and Cb (yellow dashed arrow), Scale bar, 1000 μm. (**D**) Representative co-immunofluorescence images with GFP and TWIK-1 in the DG. Scale bar, 200 μm. (**E**) Enlarged inset from D. Scale bar, 50 μm. (**F**) Representative co-immunofluorescence images with GFP and TWIK-1 in the LEC. Scale bar, 200 μm. (**G**) Enlarged inset from F. Scale bar, 50 μm. (**H**) Representative co-immunofluorescence images with GFP and TWIK-1 in the Cb. Scale bar, 200 μm. (**I**) Enlarged inset from H. Scale bar, 50 μm.

A modified BAC clone covering the TWIK-1 (KCNK1) locus (GENSAT1-BX1474, Entrez Gene ID: 16525) was purchased from the BACPAC Resources Center. As detailed in the GENSAT homepage (www.gensat.org accessed on 14 October 2021), the modified BAC clone (GENSAT1-BX1474) was generated from a BAC clone (RP23-385E8) by recombination. The modified BAC DNAs were purified using a Large-Construct Kit (Qiagen, Cat#; 12462, Germantown MD, USA) and linearized by P1-Sce1 digestion. Fertilized eggs collected from C57BL/6N females were used for pronuclear injection, and the injected eggs were transplanted into pseudo-pregnant ICR females (Orient Bio, Seongnam-si, Korea). TWIK-1 BAC-GFP Tg mice were genotyped using a pair of primers (F, 5′-CACCACGTCACCGCCACAT-3′; R, 5′-TAGCGGCTGAAGCACTGCA-3′) (Figure 1A,B). Based on the public in situ hybridization data for TWIK-1 mRNA expression in the brain of male postnatal 56 (P56) aged mice (https://mouse.brain-map.org/experiment/show/67850952, accessed on 14 October 2021), male TWIK-1 BAC-GFP Tg mice aged P56 were also used for the experiments showing GFP and TWIK-1 protein expression. All experiments were conducted in accordance with the protocols approved by the instructional guidelines of the Korea University Institutional Animal Care & Use Committee (approval number: KUIACUC-2019-0050).

### 2.2. Preparation of Brain Slices

Male TWIK-1 BAC-GFP Tg mice aged P56 were anesthetized with isoflurane and subjected to intracardiac perfusion with phosphate-buffered saline (PBS) followed by 4% paraformaldehyde (PFA). Extracted brains were fixed for 16 h with 4% PFA and then incubated for 72 h with 30% sucrose in PBS. Brains were embedded with optimal cutting temperature (OCT) compound (Sakura Finetek, Cat#; 4583, Torrance CA, USA) and frozen on dry ice. Thereafter, 30-μm-thick brain slices were obtained using a cryostat (Leica Biosystmes, RRID; SCR_018061, Richmond, IL, USA). 

### 2.3. Immunohistochemistry

Brain slices were washed with PBS for 20 min at room temperature (RT), followed by antigen retrieval with 10 mM sodium citrate buffer at 85 °C for 30 min. The slices were washed with PBS and permeablized with 0.4% Triton X-100 in PBS at RT for 20 min. Then, the slices were blocked with 10% donkey serum and 0.1% Triton X-100 in PBS at RT for 3 h followed by incubation with the primary antibodies, 5% donkey serum, and 0.1% Triton X-100 in PBS at 4 °C for 16 h. After washing with 0.1% Triton X-100 in PBS at RT for 15 min three times, the secondary antibodies, 5% donkey serum, and 0.1% Triton X-100 were placed in PBS at 4 °C for 2 h. The slices were counterstained with DAPI and mounted with VECTASHIELD antifade mounting media (Vector Laboratories, Cat#; H-1000, Burlingame CA, USA). Images were acquired with a ZEISS Axio Scan.Z1 slide scanner (Carl Zeiss, RRID; SCR_020927, Oberkochen, Germany) for macroscopic image data and a Nikon Eclipse Ti2 confocal microscope (Nikon, RRID; SCR_021068, Tokyo, Japan) for microscopic image data. The following antibodies were used: chicken anti-GFP (Abcam, Cat#; ab136970, 1:300); rabbit anti-TWIK-1 (Alomone labs, Cat#; APC-10, 1:200); mouse anti-NeuN (Abcam, Cat#; ab104224, 1:100); rabbit anti-GAD67 (GeneTex, Cat#; GTX113190, 1:300); guinea pig anti-doublecortin (Millipore, Cat#; AB2253, 1:100); rabbit anti-calbindin (Swant, Cat#; CB38, 1:2000); rat anti-Ki67 (Invitrogen, Cat#; 14-5698-85, 1:200); rat anti-GFAP (Invitrogen, Cat#; 13-0300, 1:500); rabbit anti-NG2 (Millipore, Cat#; AB5320, 1:300); and Alexa Fluor 488-, 594-, and 647-conjugated secondary antibodies (Jackson ImmunoResearch, 1:300).

### 2.4. Image Acquisition and Quantification

Macroscopic images for 16 coronal brain slices with 0.6~1 mm intervals were acquired using a ZEISS Axio Scan.Z1 slide scanner (Carl Zeiss, RRID: SCR_020927, Oberkochen, Germany) equipped with a Plan-Apochromat 10×/0.45 objective (Carl Zeiss, Oberkochen, Germany) at 16-bit depth. To quantify GFP expression in the various brain regions, we selected 13 brain regions showing the prominent GFP expression among 16 coronal brain slices. We measured GFP fluorescent intensity in 13 anatomical brain regions of interest (Table 1). Arbitrary units showing the mean GFP intensity in 13 brain regions were measured and then divided into five levels (0–1000 for +, 1000–1300 for ++, 1300–1600 for +++ and 1600–1900 for ++++, and 1900–2200 for +++++). Microscopic images for DG, LEC, and Cb were acquired using a Nikon Eclipse Ti2 confocal microscope (Nikon, RRID; SCR_021068, Tokyo, Japan) using a Plan Apo Lambda 20×/0.75 objective (Nikon, Material#: MRD00205, Tokyo, Japan).

### 2.5. Fluorescence In Situ Hybridization (FISH)

FISH was performed using an RNAscope Fluorescent Multiplex Kit (Advanced Cell Diagnostics, RRID; SCR_012481, CA, USA) and a *Kcnk1*-C1 probe (Advanced Cell Diagnostics, Cat#; ACD 535421, CA, USA) according to the manufacturer’s instructions. Subsequently, 15-μm fresh frozen brain slices were fixed with 4% PFA for 15 min and then washed in 50% (×1), 70% (×1), and 100% (×2) ethanol for 5 min each. After air drying for 5 min, the slices were digested with protease solution at RT for 30 min, followed by washing with PBS three times. The pre-warmed probe was applied to the slices, which were incubated in a humidified oven at 40 °C for 2 h. Slices were washed twice with the wash buffer and then amplified with AMP 1 to AMP 4, followed by counterstaining with DAPI. Slices were mounted with ProLong Gold Antifade (Thermo Fisher Scientific, Cat#; P36930, MA, USA). Images were acquired with a Ti2 confocal microscope (Nikon, RRID; SCR_021068, Tokyo, Japan). Z-stack images were acquired using a Nikon Eclipse Ti2 confocal microscope (Nikon, RRID; SCR_021068, Tokyo, Japan) using a Plan Apo Lambda 40x/0.95 objective (Nikon, Material#: MRD00405, Tokyo, Japan) with a 0.6232 × 0.6232 × 2 μm^3^ voxel size. Kcnk1 mRNA spots were manually counted from maximum z-projection images using Fiji software (Max Plank Institute of Molecular Cell Biology and Genetics, RRID: SCR_002285, Dresden, Germany).

### 2.6. Intraperitoneal Injection of KA

Mice were treated with an intraperitoneal injection of KA (30 mg/kg) (Milestone Pharmtech USA Inc., Cat#; 6M-0100, New Brunswick NJ, USA) emulsified in 0.9% normal saline [17]. Saline-treated mice served as controls. Five days after KA treatment, the mice were sacrificed, and the extracted brain was sampled for immunohistochemistry and FISH. 

### 2.7. Statistical Analysis

Statistical analysis was performed using GraphPad Prism 8 (GraphPad, version 8.4.3., USA) (Supplementary Materials Table S1). Experimental groups were compared using unpaired, nonparametric Student’s *t*-test. All average values are presented as Mean ± SEM. Cohen’s effect size (*d*) was calculated according to the following formula:

(1) $$d=\left({\mathrm{Mean}}_{2}-{\mathrm{Mean}}_{1}\right)÷\sqrt{\left(\frac{\left(Standard\;deviation_{1}^{\;2}+Standard\;deviation_{2}^{\;2}\right)}{2}\right)}$$

## 3. Results

### 3.1. Generation and Validation of the TWIK-1 BAC-GFP Tg Mouse Line

To examine the gene expression of TWIK-1 in the mouse brain, we generated a TWIK-1 BAC-GFP Tg mouse line using a modified BAC clone containing the GFP gene (GENSAT-BX1474). The modified BAC clone was generated by inserting GFP into a BAC clone (RP23-385E8) via homologous recombination in *Escherichia coli* [18] (www.gensat.org accessed on 14 October 2021). The modified GFP-expressing BAC construct contained almost 80–90 kb of the promoter genomic region for mouse TWIK-1 (Figure 1A). A founder line was selected by genotyping PCR with a designed primer pair (Figure 1B). Next, we validated whether GFP expression in transgenic mice was representative of endogenous TWIK-1 expression in the brain. In order to systematically analyze GFP expression in our TWIK-1 BAC-GFP Tg mouse, we built a pipeline of experiments including macroscopic to microscopic resolution (Supplementary Materials Figure S1). Immunohistochemical analysis of serial brain sections showed that GFP immunoreactive signals were highly detected in various brain regions, including DG, LEC, and Cb (Figure 1C). The GFP immunoreactive signals were properly co-localized with TWIK-1 immunoreactive signals (Figure 1D–I). All GFP-expressing cells were completely matched with TWIK-1 immunoreactive signals in the DG, LEC, and Cb (Figure 1D–G), although the GFP immunoreactive signals did not cover all TWIK-1-expressing cells.

From the microscopic analysis of GFP expression, we found that GFP immunoreactive signals were highly detected in various regions of the mouse brain (Table 1). GFP signals in the DG, reticular thalamic nucleus, and cerebellar granule layer are similar to TWIK-1 mRNA expression patterns in other previously reported studies [13,14]. In addition, we found a novel strong GFP expression in several brain regions, such as the caudate-putamen, peri-amygdala area, and LEC. Since GFP-expressing cells were also co-localized with TWIK-1 immunoreactive signals, we concluded that the TWIK-1 BAC-GFP Tg mouse could be a useful animal for providing fundamental information on TWIK-1 expression in various regions of the mouse brain.

### 3.2. TWIK-1 Expression in Excitatory Neurons in the DG, LEC, and Cb

**Figure 2:**
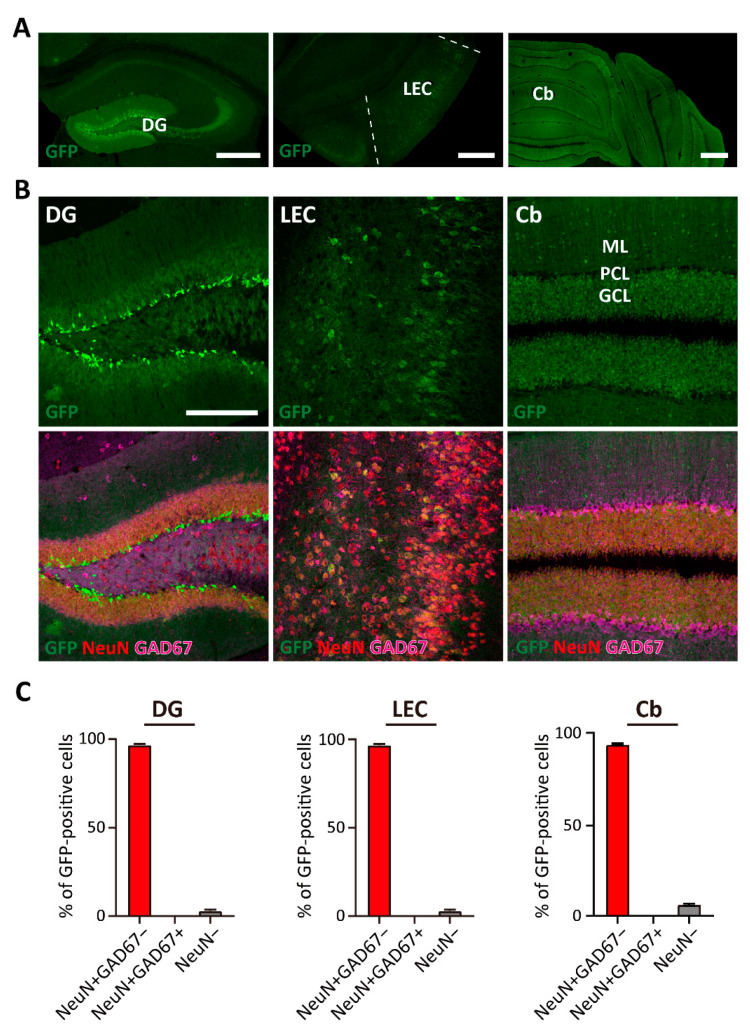
Cellular identification of GFP-expressing cells of the DG, LEC, and Cb in P56 of TWIK-1 BAC-GFP Tg mice. (**A**) Overview of GFP expression in DG, LEC, and CB of TWIK-1 BAC-GFP Tg mice. Scale bar, 200 μm. (**B**) Representative co-immunofluorescence images with GFP, NeuN, and GAD67 antibodies. Scale bar, 200 μm. (**C**) Quantification bar graph of the cell type of GFP-positive cells in each brain area from B. Quantification was analyzed by the percentage of each cell type from all GFP-positive cells. Raw data are listed in Supplementary Materials Table S1. Data are presented as the Mean ± SEM.

Among the brain regions showing strong GFP signals (Table 1), we further analyzed TWIK-1 expression in the hippocampus, LEC, and Cb (Figure 2A). In the hippocampus, we found strong GFP expression in the granule cell layer of the DG, while relatively low GFP expression was detected in the pyramidal layer of Cornu Ammonis area 1 (CA1) (Figure 2A, left column). Using the neuronal marker NeuN and inhibitory neuronal marker GAD67, we examined the cell type of GFP-positive cells in the DG and found almost all GFP-positive cells were excitatory neurons in the DG (Figure 2B,C, left column). We also found numerous GFP-expressing cells located at the LEC, which is one of the major input sources for the DG (Figure 2A, middle column). Most of these cells were also identified as excitatory cells, which represent pyramidal neurons in LECs expressing GFP at relatively high levels (Figure 2B,C, middle column). At the Cb, GFP expression was predominantly found in the granule cell layer of whole cerebellar regions (Figure 2A, right column). Similar to TWIK-1 expression in the DG and LEC, most TWIK-1-expressing cells were excitatory neurons (Figure 2B,C, right column). 

### 3.3. TWIK-1 Highly Expressed in the Immature Neurons of the DG 

**Figure 3:**
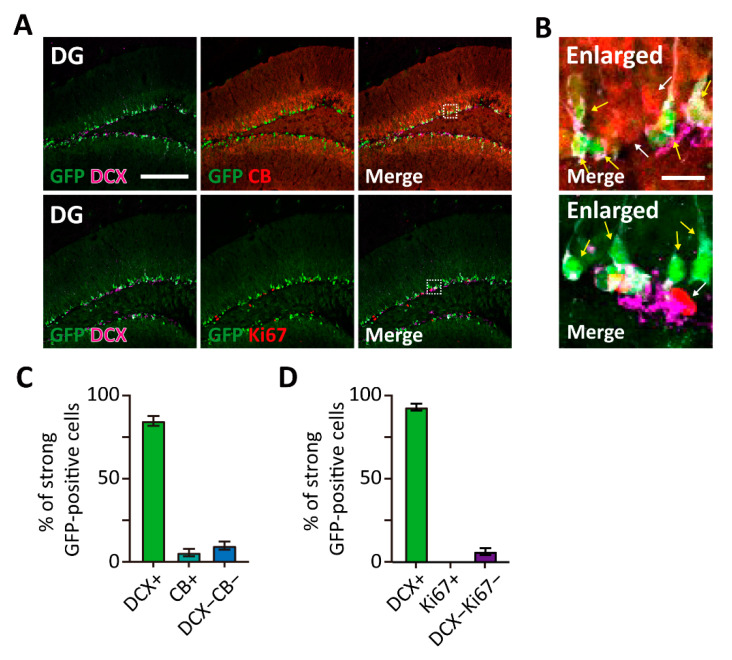
High TWIK-1 expression in immature neurons of the DG in TWIK-1 BAC-GFP Tg mice. (**A**) Representative co-immunofluorescence images with GFP, DCX, CB, and Ki67 in P56 of DG. Scale bar, 200 μm. (**B**) Enlarged inset from A. Most of strong GFP-expressing cells co-labeled with DCX (yellow arrow), but not with CB and Ki67 (white arrow). Scale bar, 10 μm. (**C**,**D**) Quantification of the cell type of strong GFP-expressing cells. Raw data are listed in Supplementary Materials Table S1. Data are presented as the Mean ± SEM.

As shown in Figure 2A, strong GFP-expressing cells were mainly localized at the boundary of the hilus and granule cell layer in the hippocampus. Since adult neurogenesis occurs in these regions, we decided to identify the cell type of strong GFP-expressing cells. Immunohistochemical analysis was performed with several neurogenesis markers, such as Ki67 for proliferating cells, doublecortin (DCX) for immature granule cells, and calbindin (CB) for mature granule cells (Figure 3A,B). Notably, strong GFP-expressing cells were mostly co-labeled with the immature neuronal marker DCX (Figure 3C,D). Moreover, no or few of GFP-expressing cells were co-labeled with CB and Ki67. Our results indicate that TWIK-1 is highly expressed in dentate granule cells, especially in immature granule cells.

### 3.4. Glial Expression of TWIK-1 in the DG

**Figure 4:**
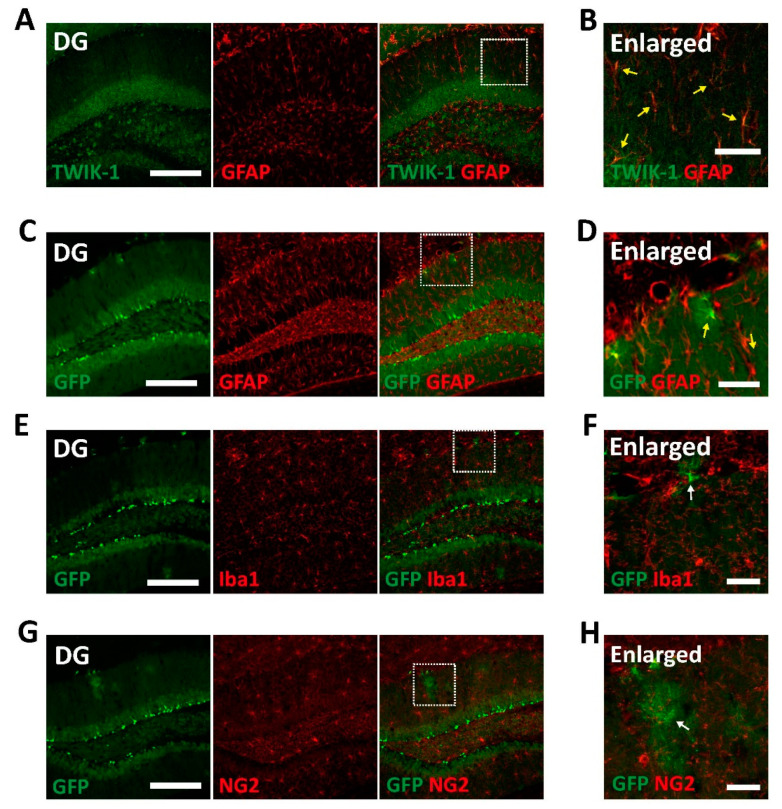
Glial expression of TWIK-1 in TWIK-1 BAC-GFP Tg mice. (**A**) Representative co-immunofluorescence images with TWIK-1 and GFAP. Scale bar, 200 μm. (**B**) Enlarged inset from A. Yellow arrow indicates double immunoreactive cells with TWIK-1 and GFAP. Scale bar, 50 μm. (**C**) Representative co-immunofluorescence images with GFP and GFAP. Scale bar, 200 μm. (**D**) Enlarged inset from C. Yellow arrow indicates double immunoreactive cells with GFP and GFAP. Scale bar, 50 μm. (**E**) Representative co-immunofluorescence images with GFP and Iba1. Scale bar, 200 μm. (**F**) Enlarged inset from E. There are no Iba1-positive glial-like GFP-expressing cells (White arrow). Scale bar, 50 μm. (**G**) Representative co-immunofluorescence images with GFP and NG2. Scale bar, 200 μm. (**H**) Enlarged inset from G. There are no NG2-positive glial-like GFP-expressing cells (White arrow). Scale bar, 50 μm.

Besides the neuronal expression of TWIK-1, it has been reported that TWIK-1 is also expressed in astrocytes [9,10,11,12,19]. Therefore, we next examined GFP signals in non-neuronal cells in the brains of TWIK-1 BAC-GFP Tg mice. Endogenous TWIK-1s were expressed in almost all glial fibrillary acidic protein (GFAP)-positive astrocytes (Figure 4A,B). Additionally, GFP-expressing non-neuronal cells were identified as GFAP-positive astrocytes but not ionized calcium binding adaptor molecule 1 (Iba1)-positive microglia or neural/glial antigen 2 (NG2)-positive oligodendrocyte cells (Figure 4C–H). These data also showed that all GFP-expressing astrocytes were completely matched with TWIK-1 immunoreactive signals, although the GFP immunoreactive signals did not include all TWIK-1-expressing cells.

### 3.5. Upregulation of TWIK-1 Expression in the Intraperitoneal KA-Injected Hippocampus

**Figure 5:**
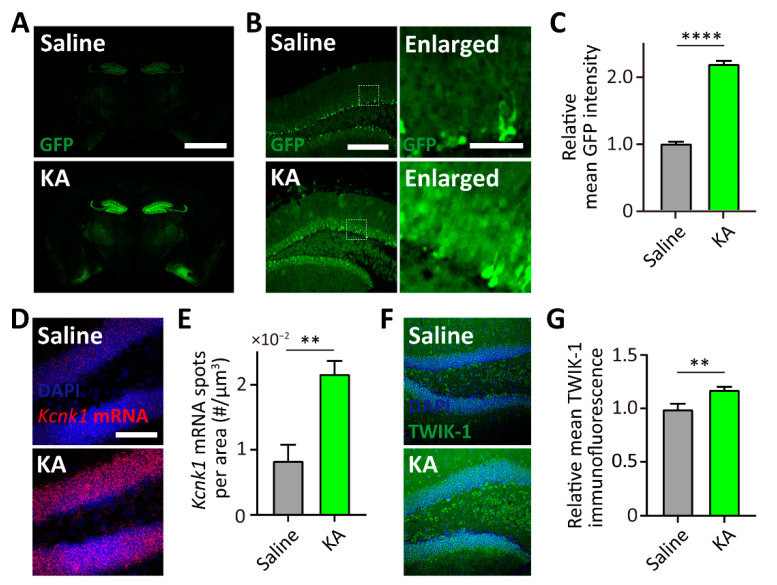
TWIK-1 BAC-GFP Tg mice represent kainic acid (KA)-induced increase of TWIK-1 expression. (**A**) Representative GFP expression in a brain slice from saline or KA-treated TWIK-1 BAC-GFP Tg mice. Scale bar, 1000 μm. (**B**) Representative GFP expression in the DG from saline or KA-treated TWIK-1 BAC-GFP Tg mice. Scale bar, 200 μm. Enlarged inset from the experiment. Scale bar, 50 μm. (**C**) Quantification of relative mean GFP intensity of the granule cell layer. (**D**) Representative *Kcnk1* mRNA fluorescence in situ hybridization (FISH) images of DG from saline- or KA-treated mice. Scale bar, 100 μm. (**E**) Quantification bar graph of *Kcnk1* mRNA spot density in the granule cell layer from saline- or KA-treated mice. (**F**) Representative TWIK-1 immunofluorescence images of DG from saline- or KA-treated mice. Scale bar, 200 μm. (**G**) Quantification bar graph of the relative mean TWIK-1 immunofluorescence in the granule cell layer of saline- or KA-treated mice. Raw data are listed in Supplementary Materials Table S1. **** *p* < 0.0001; ** *p* < 0.01, two-tailed *t* tests. Data are presented as the Mean ± SEM.

In general, BAC transgenic mice are one of the most used animal models for the correlation of gene expression due to modified BACs carrying a large-sized promoter [15,16]. Since a previous study reported that TWIK-1 channel proteins are elevated when KA was directly injected into the hippocampus [20], we next determined whether our TWIK-1 BAC-GFP Tg mouse can mimic the KA-induced upregulation of TWIK-1 expression in the hippocampus. To examine the effects of KA on the GFP and endogenous TWIK-1 expression, saline or KA were intraperitoneal injected into the TWIK-1 BAC-GFP Tg mice. As shown in Figure 5A,B, 5 days after KA injection, GFP expression was dramatically increased in the hippocampal DG. Additionally, we found that the GFP expression increased with number of astrocytes (Figure 5A,B). To confirm elevated endogenous TWIK-1 expression in the hippocampal DG by KA injection, we performed a FISH experiment with a probe for TWIK-1 mRNA and immunohistochemistry analysis with an antibody against TWIK-1 channel protein. As expected, KA injection resulted in an increase of TWIK-1 mRNA and protein levels in the hippocampal DG (Figure 5C–F). Taken together, these results indicate that our TWIK-1 BAC-GFP Tg mouse represents KA-induced upregulation of TWIK1 in the DG.

## 4. Discussion

It has been reported that TWIK-1 plays important roles in DG granule cells and astrocytes in the hippocampus [7,8,9,10,11]. Despite high-level TWIK-1 expression in various brain regions, the physiological roles of TWIK-1 in other brain regions remain elusive due to the lack of information on TWIK-1 expression in the brain. Here, we generated and characterized TWIK-1 BAC-GFP Tg mice, which are a useful animal model that can monitor TWIK-1 gene expression (Figure 1 and Table 1). Our data clearly showed that GFP signals in the TWIK-1 BAC-GFP Tg mouse represent profiles of TWIK-1 expression in various brain regions at cell-type specific resolution (Figure 2, Figure 3 and Figure 4). In addition, our data show that TWIK-1 BAC-GFP Tg mice can mimic the upregulation of TWIK-1 expression in hippocampal DG granule neurons following systemic KA injection (Figure 5). 

Our transgenic mice show that granule neurons in both DG and Cb express TWIK-1 at relatively higher levels than other TWIK-1-expressing regions (Figure 1 and Table 1). These results are consistent with previous reports on the functional existence of TWIK-1 in granule neurons of the DG and Cb [7,8,21]. As our previous studies showed that deficiency of TWIK-1 increases RMP in DG granule neurons [7,8], it is also possible that TWIK-1 can contribute to the regulation of RMP in cerebellar granule neurons, although further studies are required. Since alteration of RMP was involved in the maturation process of postnatally developing cerebellar granule neurons [22] and consolidation of long-term phase learning of the vestibule-ocular reflex [23], TWIK-1 might contribute to the physiological functions of cerebellar granule neurons via regulation of RMP. Physiological or pathophysiological functions of TWIK-1 in cerebellar granule neurons should be studied in future studies. 

TWIK-1 expression was highest in immature neurons of the DG (Figure 3). Immature granule neurons have a distinct high RMP, which makes them have hyper-excitable electrophysiological properties [24]. Since a deficiency in TWIK-1 slightly increases RMP in DG mature granule neurons [7,8], high TWIK-1 expression in immature granule neurons might be a key regulator for maintaining distinctly high RMP of immature granule neurons. Since hyperpolarization of RMP was also examined during the maturation of cerebellar granule neurons [22] and TWIK-1 was highly expressed in the cells (Figure 2B), it is plausible that TWIK-1 could play a critical role in the maturation of cerebellar granule neurons. To verify this hypothesis, it will be necessary to study TWIK-1 function in the maturation of DG and Cb granule neurons with gain- or loss-of-function approaches.

We found strong TWIK-1 expression in the hippocampal DG and LEC, which are important brain regions for memory formation [25,26]. As mentioned above, a decrease in TWIK-1 expression makes neurons excitable; therefore, it could disturb activation of a subset of granule cells that are required for the acquisition of new memories. There is still no evidence for this idea; however, it was reported that deficiency of TASK-3, a member of the K2P family, perturbs circuit activity in the DG [27]. Interestingly, it has been reported that TWIK-1 can make the heterodimeric channel with TASK-3 in granule cells of the DG [7,8]. Therefore, it is also plausible that TWIK-1 deficiency may be involved in perturbation of circuit activity in granule cells of the DG. In addition, TWIK-1 is downregulated in the hippocampus of patients with Alzheimer’s disease [28,29], which is a well-known memory-related neurodegenerative disorder. Based on the above, it is necessary to confirm the relationship between TWIK-1 expression and the functions of the hippocampal memory circuit.

We also found that KA injection increased TWIK-1 expression at both the mRNA and protein levels, and our TWIK-1 BAC-GFP Tg mice could be used to properly measure changes in TWIK-1 expression (Figure 5), which is consistent with a previous report [20]. Meanwhile, KA injection is popularly used as a general temporal lobe epilepsy model in rodents [30]. Therefore, increased expression of TWIK-1 might be a consequence of the defense mechanism against epileptic seizures induced by KA injection. Since activators of potassium channels are known to be used as drugs for epilepsy [31,32], it seems that TWIK-1 could be a novel therapeutic target for epilepsy and TWIK-1 functions in epilepsy should be examined in future studies.

Our results showed that TWIK-1 BAC-GFP Tg mice can be used as an animal model for studying the TWIK-1 functions in the brain. However, the Tg mice still have some limitations to represent the endogenous TWIK-1 expression in the brain. There are gene expression data regarding the expression of TWIK-1 in astrocytes [33,34,35], and our previous reports also showed that TWIK-1 expression in hippocampal astrocytes [9,11]. Contrary to these results, only a restricted number of astrocytes expressed GFP in our transgenic mice (Figure 4). In addition, the GFP immunoreactive signals did not cover all endogenous TWIK-1-expressing neurons (Figure 1D–G). These limitations of our TWIK-1 BAC-GFP Tg mice implied that a larger TWIK-1 promoter size is required to mimic endogenous TWIK-1 expression in astrocytes, despite the relatively large size of TWIK-1 promoter in the modified BAC clone used in this study. 

In conclusion, the TWIK-1 BAC-GFP Tg mouse generated in this study could be a useful animal model for the study of the molecular and cellular mechanisms of TWIK-1 expression and may provide opportunities to elucidate new functions of TWIK-1 in a variety of brain regions.

## Figures and Tables

**Table 1:** GFP expression in various brain regions of the TWIK-1 BAC-GFP Tg mice.

Brain Region	Expression
Outer plexiform layer	+
Piriform cortex	++
Caudate putamen	+++
Hypothalamus	+
Dentate gyrus	+++++
Reticular thalamic nucleus	+++
Peri-amygdala area	++++
Lateral entorhinal cortex	++++
Lateral lemniscus	+++
Reticular trigeminal nucleus	+++
Superior olivary complex	+++
Cerebellar granule cell layer	+++++
Cerebellar dentate nucleus	+++

## Data Availability

Not applicable.

## Supplementary Materials

The following are available online at https://www.mdpi.com/article/10.3390/cells10102751/s1, Figure S1: Pipeline scheme for analysis of GFP expression in TWIK-1 BAC-GFP Tg mice.

## References

[1] Enyedi P., Czirjak G. (2010). Molecular background of leak K+ currents: Two-pore domain potassium channels. Physiol. Rev..

[2] Lesage F., Guillemare E., Fink M., Duprat F., Lazdunski M., Romey G., Barhanin J. (1996). TWIK-1, a ubiquitous human weakly inward rectifying K+ channel with a novel structure. EMBO J..

[3] Rajan S., Plant L.D., Rabin M.L., Butler M.H., Goldstein S.A. (2005). Sumoylation silences the plasma membrane leak K+ channel K2P1. Cell.

[4] Feliciangeli S., Bendahhou S., Sandoz G., Gounon P., Reichold M., Warth R., Lazdunski M., Barhanin J., Lesage F. (2007). Does sumoylation control K2P1/TWIK1 background K+ channels?. Cell.

[5] Chatelain F.C., Bichet D., Douguet D., Feliciangeli S., Bendahhou S., Reichold M., Warth R., Barhanin J., Lesage F. (2012). TWIK1, a unique background channel with variable ion selectivity. Proc. Natl. Acad. Sci. USA.

[6] Nie X., Arrighi I., Kaissling B., Pfaff I., Mann J., Barhanin J., Vallon V. (2005). Expression and insights on function of potassium channel TWIK-1 in mouse kidney. Pflug. Arch..

[7] Yarishkin O., Lee D.Y., Kim E., Cho C.H., Choi J.H., Lee C.J., Hwang E.M., Park J.Y. (2014). TWIK-1 contributes to the intrinsic excitability of dentate granule cells in mouse hippocampus. Mol. Brain.

[8] Choi J.H., Yarishkin O., Kim E., Bae Y., Kim A., Kim S.C., Ryoo K., Cho C.H., Hwang E.M., Park J.Y. (2018). TWIK-1/TASK-3 heterodimeric channels contribute to the neurotensin-mediated excitation of hippocampal dentate gyrus granule cells. Exp. Mol. Med..

[9] Hwang E.M., Kim E., Yarishkin O., Woo D.H., Han K.S., Park N., Bae Y., Woo J., Kim D., Park M. (2014). A disulphide-linked heterodimer of TWIK-1 and TREK-1 mediates passive conductance in astrocytes. Nat. Commun..

[10] Cho C.H., Hwang E.M., Park J.Y. (2017). Emerging Roles of TWIK-1 Heterodimerization in the Brain. Int. J. Mol. Sci..

[11] Bae Y., Choi J.H., Ryoo K., Kim A., Kwon O., Jung H.G., Hwang E.M., Park J.Y. (2020). Spadin Modulates Astrocytic Passive Conductance via Inhibition of TWIK-1/TREK-1 Heterodimeric Channels. Int. J. Mol. Sci..

[12] Ryoo K., Park J.Y. (2016). Two-pore Domain Potassium Channels in Astrocytes. Exp. Neurobiol..

[13] Talley E.M., Solorzano G., Lei Q., Kim D., Bayliss D.A. (2001). Cns distribution of members of the two-pore-domain (KCNK) potassium channel family. J. Neurosci..

[14] Aller M.I., Wisden W. (2008). Changes in expression of some two-pore domain potassium channel genes (KCNK) in selected brain regions of developing mice. Neuroscience.

[15] Heintz N. (2001). BAC to the future: The use of bac transgenic mice for neuroscience research. Nat. Rev. Neurosci..

[16] Yang X.W., Gong S. (2005). An overview on the generation of BAC transgenic mice for neuroscience research. Curr. Protoc. Neurosci..

[17] Xue F., Shi C., Chen Q., Hang W., Xia L., Wu Y., Tao S.Z., Zhou J., Shi A., Chen J. (2017). Melatonin Mediates Protective Effects against Kainic Acid-Induced Neuronal Death through Safeguarding ER Stress and Mitochondrial Disturbance. Front. Mol. Neurosci..

[18] Gong S., Zheng C., Doughty M.L., Losos K., Didkovsky N., Schambra U.B., Nowak N.J., Joyner A., Leblanc G., Hatten M.E. (2003). A gene expression atlas of the central nervous system based on bacterial artificial chromosomes. Nature.

[19] Cahoy J.D., Emery B., Kaushal A., Foo L.C., Zamanian J.L., Christopherson K.S., Xing Y., Lubischer J.L., Krieg P.A., Krupenko S.A. (2008). A transcriptome database for astrocytes, neurons, and oligodendrocytes: A new resource for understanding brain development and function. J. Neurosci..

[20] Young C.C., Stegen M., Bernard R., Muller M., Bischofberger J., Veh R.W., Haas C.A., Wolfart J. (2009). Upregulation of inward rectifier K+ (Kir2) channels in dentate gyrus granule cells in temporal lobe epilepsy. J. Physiol..

[21] Plant L.D., Zuniga L., Araki D., Marks J.D., Goldstein S.A. (2012). SUMOylation silences heterodimeric TASK potassium channels containing K2P1 subunits in cerebellar granule neurons. Sci. Signal..

[22] Makoto O., Haruka A., Michiko K., Kouichirou I., Tatsuto K., Shigetada N. (2009). Role of Calcineurin Signaling in Membrane Potential-Regulated Maturation of Cerebellar Granule Cells. J. Neurosci..

[23] Patricia S., Martijn S., Guillermo S., Aleksandra B., Ilse K., York R., William W., Christian A.H., Chris I.D.Z., Thomas J.J. (2012). Raising cytosolic Cl− in cerebellar granule cells affects their excitability and vestibulo-ocular learning. EMBO J..

[24] Liu Y.B., Lio P.A., Pasternak J.F., Trommer B.L. (1996). Developmental changes in membrane properties and postsynaptic currents of granule cells in rat dentate gyrus. J. Neurophysiol..

[25] Woods N.I., Vaaga C.E., Chatzi C., Adelson J.D., Collie M.F., Perederiy J.V., Tovar K.R., Westbrook G.L. (2018). Preferential Targeting of Lateral Entorhinal Inputs onto Newly Integrated Granule Cells. J. Neurosci..

[26] Hainmueller T., Bartos M. (2020). Dentate gyrus circuits for encoding, retrieval and discrimination of episodic memories. Nat. Rev. Neurosci..

[27] Goutierre M., Al Awabdh S., Donneger F., Francois E., Gomez-Dominguez D., Irinopoulou T., Menendez de la Prida L., Poncer J.C. (2019). KCC2 Regulates Neuronal Excitability and Hippocampal Activity via Interaction with Task-3 Channels. Cell Rep..

[28] Izadi F., Soheilifar M.H. (2018). Exploring Potential Biomarkers Underlying Pathogenesis of Alzheimer’s Disease by Differential Co-expression Analysis. Avicenna J. Med. Biotechnol..

[29] Hokama M., Oka S., Leon J., Ninomiya T., Honda H., Sasaki K., Iwaki T., Ohara T., Sasaki T., LaFerla F.M. (2014). Altered expression of diabetes-related genes in Alzheimer’s disease brains: The Hisayama study. Cereb. Cortex.

[30] Levesque M., Avoli M. (2013). The kainic acid model of temporal lobe epilepsy. Neurosci. Biobehav. Rev..

[31] Kohling R., Wolfart J. (2016). Potassium Channels in Epilepsy. Cold Spring Harb. Perspect. Med..

[32] Weisenberg J.L., Wong M. (2011). Profile of ezogabine (retigabine) and its potential as an adjunctive treatment for patients with partial-onset seizures. Neuropsychiatr. Dis. Treat..

[33] Zhang Y., Chen K., Sloan S.A., Bennett M.L., Scholze A.R., O’Keeffe S., Phatnani H.P., Guarnieri P., Caneda C., Ruderisch N. (2014). An RNA-sequencing transcriptome and splicing database of glia, neurons, and vascular cells of the cerebral cortex. J. Neurosci..

[34] He L., Vanlandewijck M., Mae M.A., Andrae J., Ando K., Del Gaudio F., Nahar K., Lebouvier T., Lavina B., Gouveia L. (2018). Single-cell RNA sequencing of mouse brain and lung vascular and vessel-associated cell types. Sci. Data.

[35] Vanlandewijck M., He L., Mae M.A., Andrae J., Ando K., Del Gaudio F., Nahar K., Lebouvier T., Lavina B., Gouveia L. (2018). A molecular atlas of cell types and zonation in the brain vasculature. Nature.

