# Supplementary material for: TWIK-1 BAC-GFP Transgenic Mice, an Animal Model for TWIK-1 Expression
Source: Cells. 2021 Oct 14;10(10):2751. doi: 10.3390/cells10102751 (PMC8534699; doi:10.3390/cells10102751)
Supplement: Supplementary file 1 [file cells-10-02751-s001.zip › Ssupplementary Figure S1.pdf]

cells

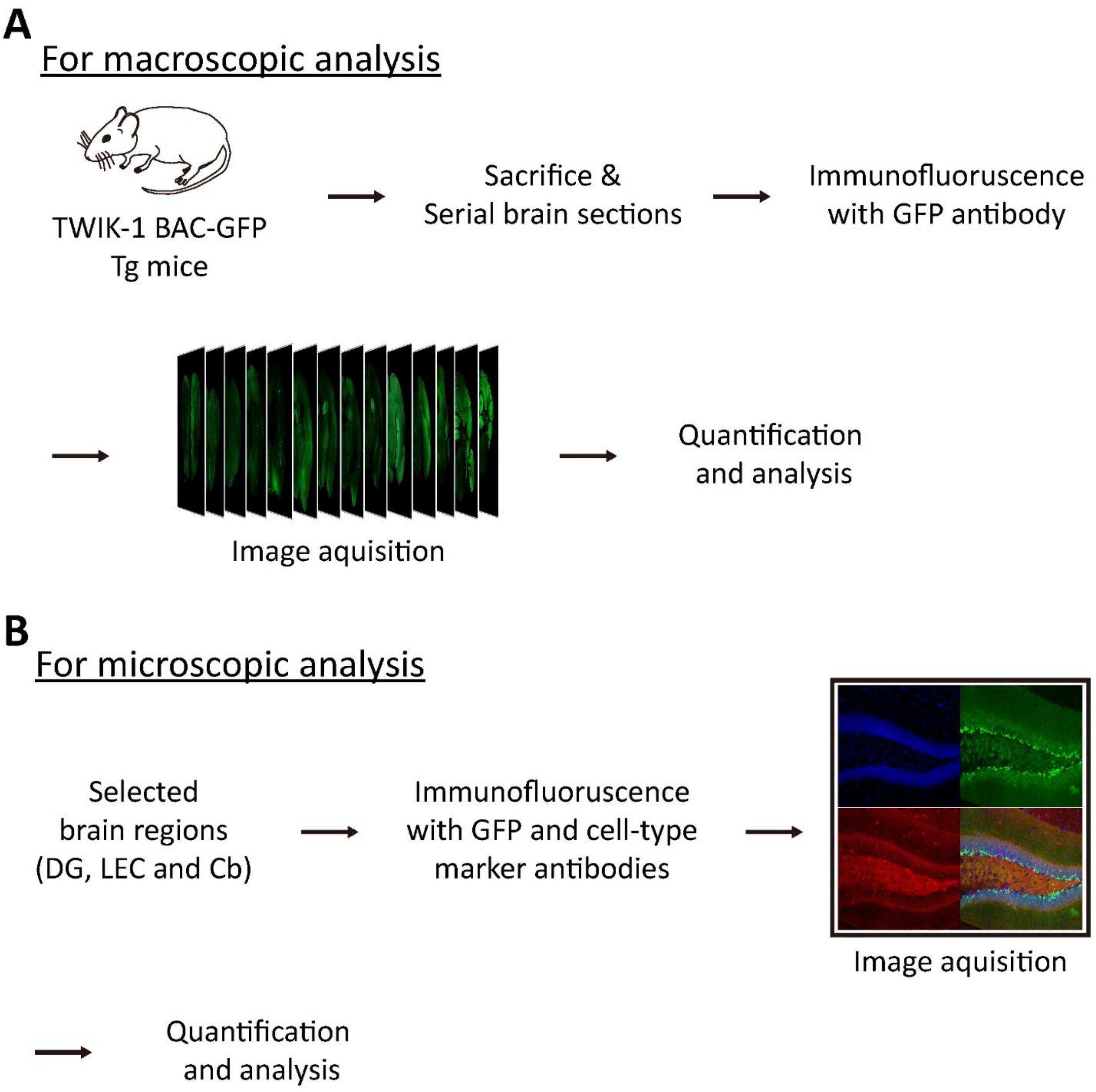

Supplementary Figure S1. Pipeline scheme for analysis of GFP expression in TWIK-1 BAC-GFP Tg mice. (**A**) TWIK-1 BAC-GFP Tg mice were sacrificed and sampled via serial coronal brain sections. Next, brain sections were immunostained with anti-GFP antibody and imaged at macroscopic resolution. GFP expressions in various brain regions were quantified and analysed. (**B**) Selected strong GFP-expressing brain regions such as DG, LEC and Cb were immunostained with cell-type specific marker antibodies and imaged at microscopic resolution. Cell-type of GFP-positive cells were analysed and quantified.
